# Successful management of thrombocytopenia by partial splenic embolization in patients with advanced gastric cancer and invasion of the splenic vein: Case reports

**DOI:** 10.1097/MD.0000000000026651

**Published:** 2021-07-16

**Authors:** Ryosuke Nakatsubo, Yoshiya Yamauchi, Taisho Hiraizumi, Fumi Naruse, Ryoya Kanda, Yuka Suzuki, Tatsuya Kakegawa, Takashi Kurosawa, Yu Yoshimasu, Toru Saguchi, Atsushi Sofuni, Takao Itoi

**Affiliations:** a Department of Gastroenterology and Hepatology, Tokyo Medical University, Tokyo, Japan; b Department of Radiology, Tokyo Medical University, Tokyo, Japan.

**Keywords:** gastric cancer, partial splenic embolization, splenic vein invasion, thrombocytopenia

## Abstract

**Rationale:**

Hypersplenism causes thrombocytopenia, which may lead to the reduction or discontinuation of chemotherapy. Partial splenic embolization (PSE) is an effective treatment for thrombocytopenia associated with hypersplenism. However, there have been no reports of patients with gastric cancer who have resumed and continued chemotherapy after PSE for splenic hypersplenism associated with tumor infiltration.

Here, we report two cases in which we performed PSE for hypersplenism associated with gastric cancer that had invaded the splenic vein. Chemotherapy was continued in both cases.

**Patient concerns:**

Both patients developed thrombocytopenia with splenomegaly due to advanced gastric cancer that required discontinuation of chemotherapy.

**Diagnosis:**

Upper gastrointestinal endoscopy and computed tomography showed advanced gastric cancer with invasion of the splenic vein and splenomegaly. Both patients developed thrombocytopenia.

**Interventions:**

Patients were treated with PSE.

**Outcomes:**

PSE produced an increase in thrombocyte count, and chemotherapy could be resumed.

**Lessons:**

PSE seems to be a useful treatment for thrombocytopenia with splenomegaly associated with advanced gastric cancer and may allow continuation of chemotherapy.

## 1 Introduction

Causes of hypersplenism include cirrhosis, obstruction of portal venous system, and splenic vein invasion. Hypersplenism causes thrombocytopenia, which may lead to the reduction or discontinuation of chemotherapy. Treatment options for splenic hyperplasia include splenectomy and partial splenic embolization (PSE). However, splenectomy is highly invasive and has a high risk of complications.^[1]^ Moreover, in cancer patients who are poor condition, splenectomy is often contra-indicated. PSE may be an alternative treatment option for thrombocytopenia with hypersplenism. ^[2,3]^ PSE is also presumed to be useful in hypersplenism, resulting from the infiltration of a tumor into the splenic vein.

However, there have been no reports of patients with gastric cancer who have resumed and continued chemotherapy after PSE for splenic hypersplenism associated with tumor infiltration.

## 2 Case presentations

### 2.1 Case 1

**Figure 1:**
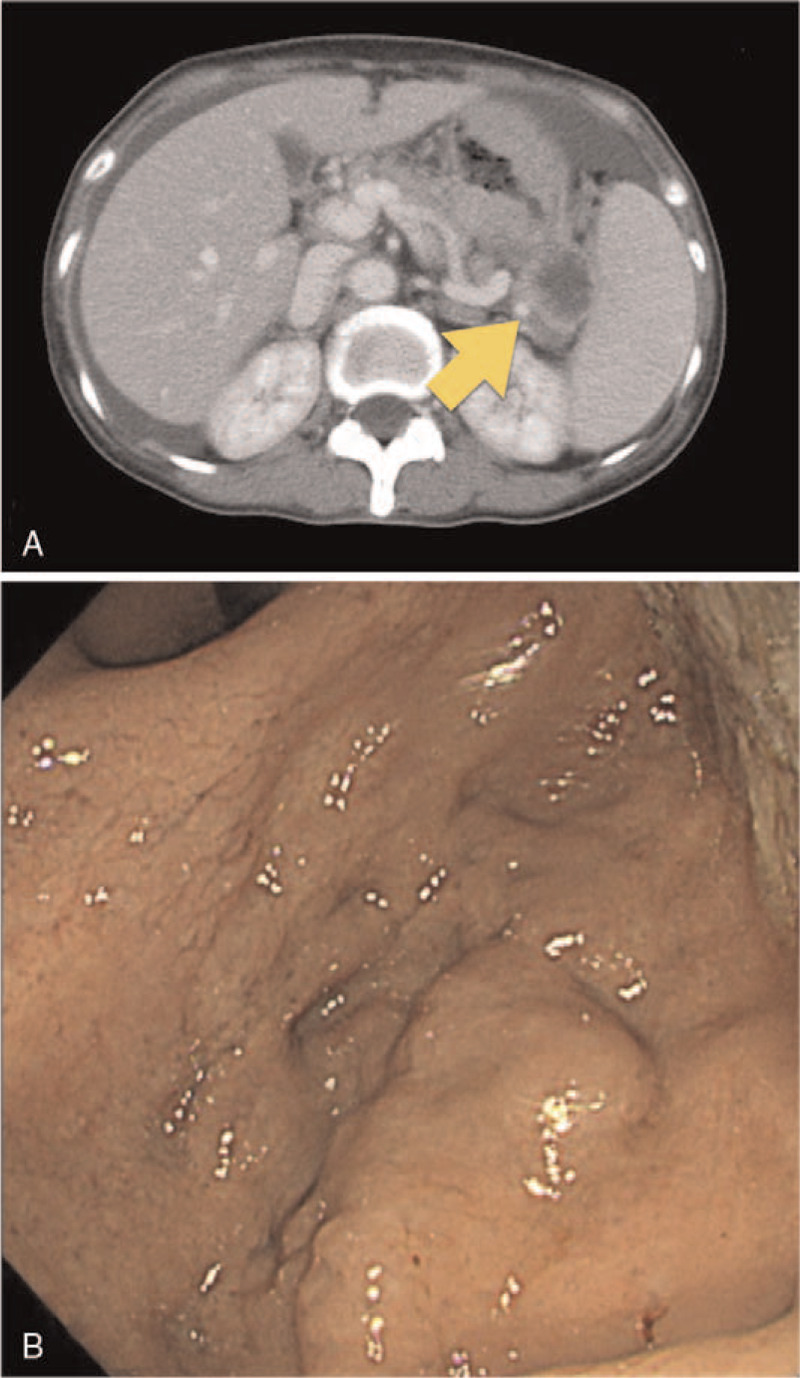
(A) Abdominal computed tomography scan showing obstruction of the splenic hilum by the tumor (arrow) in case 1. (B) Upper gastrointestinal endoscopic image showing isolated varices in the gastric fundus.

A 69-year-old female patient with a history of asthma was referred to our hospital for investigation of unexplained weight loss. Upper gastrointestinal endoscopy and contrast-enhanced computed tomography (CT) showed advanced gastric cancer with invasion of the splenic hilum, splenomegaly, and gastric varices. The diagnosis was cT4bN3M1stage IV-b gastric cancer (Fig. 1) The formula based on age and body weight applied estimated the splenic volume to be 78.3 ml. However, the actual splenic volume gained by the volumetric software on CT was 286 ml. There was an increasing amount of ascites and rapid tumor growth was also suspected. Bleeding from the tumor could not be controlled, and the patient required frequent blood transfusions. Palliative radiotherapy (30 Gy in 10 fractions) was administered. After hemostasis was achieved, the S-1 and oxaliplatin protocol was started. The platelets count before chemotherapy was 179.000/mm^2^. 2 months after starting chemotherapy, the platelet count decreased to 58.000/mm^2^. TS-1 was started as a second-line treatment. However, thrombocytopenia progressed, and upper gastrointestinal endoscopy revealed enlarged varices. PSE was performed because of concerns about the risk of bleeding from the gastric varices, leading to discontinuation of chemotherapy.

After careful skin preparation and local anesthesia, a femoral approach was used. The right femoral artery was punctured, and a catheter was advanced through the celiac artery to the splenic artery. Splenic arteriography was performed to confirm the branching status and plan the extent of embolization. The microcatheter was then advanced to the selective branch, which was embolized using gelatin sponge fragments.

**Figure 2:**
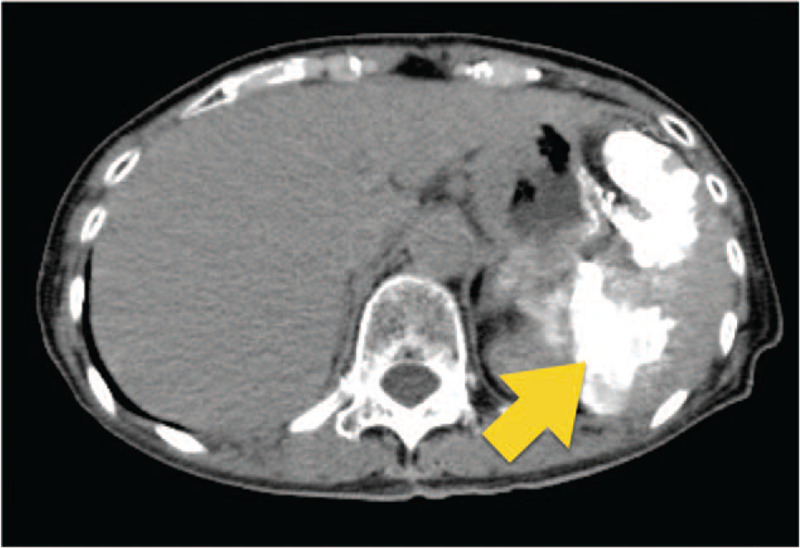
A computed tomography scan with a high absorbed area (arrow) representing an area of splenic embolization. Approximately 70% of the splenic parenchyma is ischemic.

70% embolization was achieved with no serious adverse events. (Fig. 2). The patient had mild fever for 5 days after treatment, which improved with conservative treatment.

**Figure 3:**
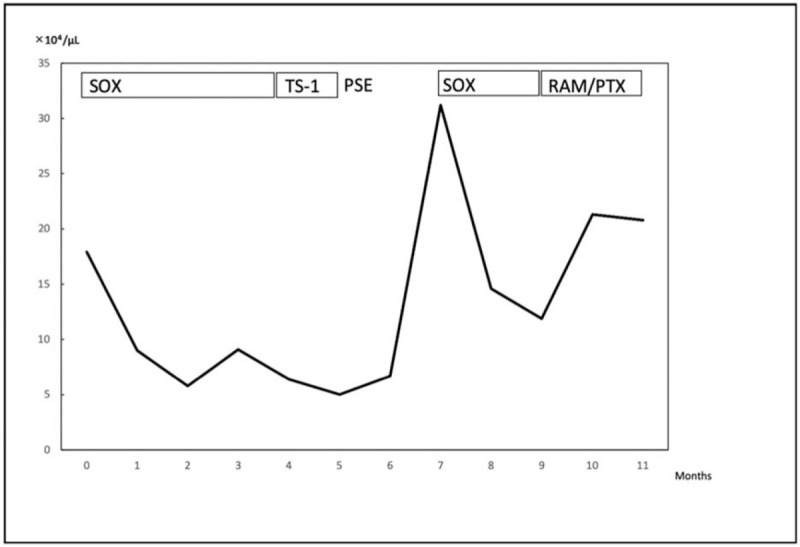
Clinical course and changes in platelet count in case 1. Platelet count increased significantly after partial splenic embolization and peaked at 310,000/mm^2^. PSE, partial splenic embolization; PTX, paclitaxel; RAM, ramucirumab; SOX, S-1 and oxaliplatin.

The hospital stay was 15 days. 2 weeks after PSE, S-1 and oxaliplatin (SOX) therapy was restarted after the varices had decreased, and the platelet count had increased from 50.000/mm^2^ to 310.000/mm^2^. Ramucirumab-paclitaxel was started as a third-line treatment because the patient developed ascites 4 months after PSE. 6 months after PSE, no thrombocytopenia was observed and chemotherapy continued (Fig. 3). However, the patient died due to progression of the primary disease.

### 2.2 Case 2

**Figure 4:**
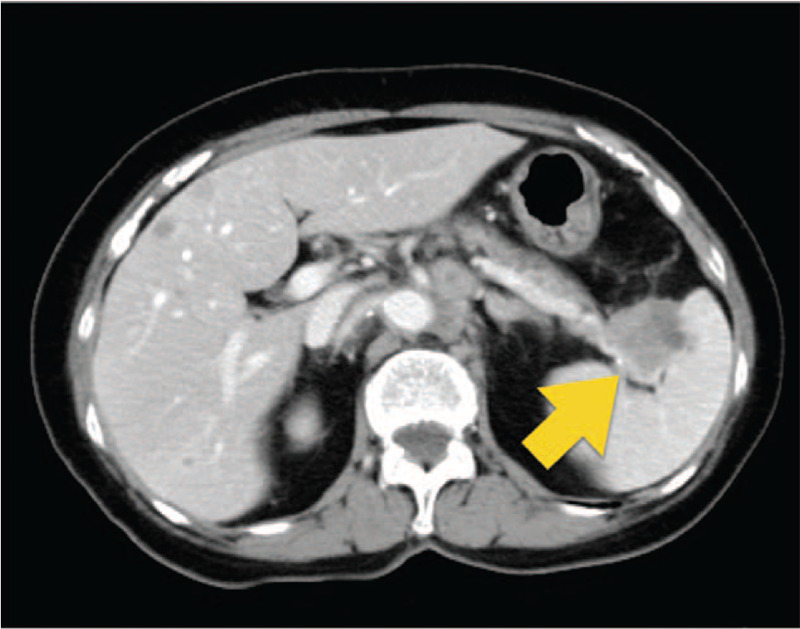
Abdominal computed tomography scan showing obstruction of the splenic vein by the tumor in case 2.

**Figure 5:**
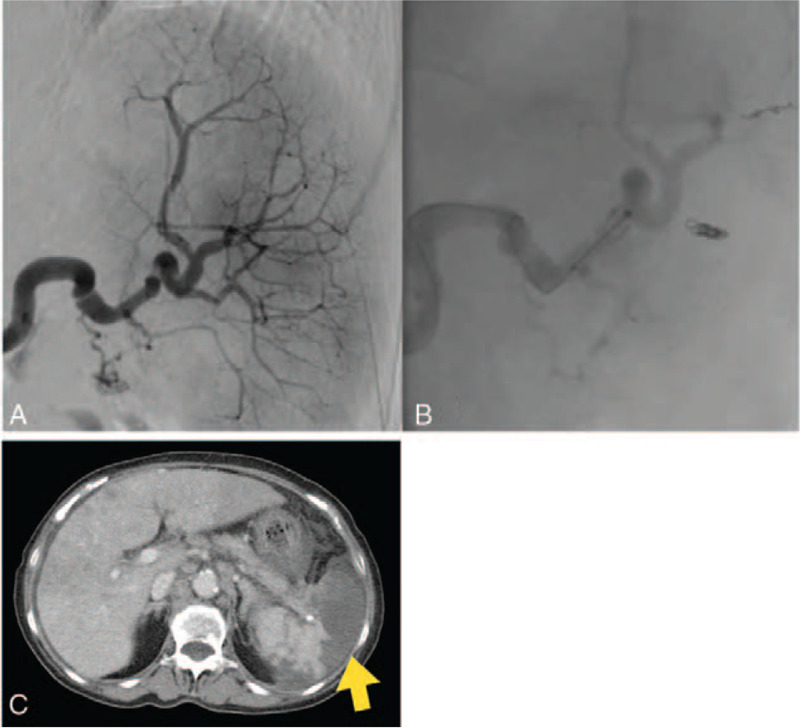
Selective arteriogram shows appearance before embolization. Post-PSE angiographic image showing 60% embolization using embospheres and microcoils performed in case 2. (C) Contrast-enhanced computed tomography scan acquired during portal phase showing splenomegaly with a large unenhanced area of the liquid (arrow) representing splenic necrosis. Approximately 60% of splenic parenchyma is ischemic.

A 75-year-old woman with Diabetes mellitus, hypertension, hepatitis C (sustained virological response) was referred to our hospital for the examination of a mass in the tail of the pancreas. Upper gastrointestinal endoscopy and CT showed advanced gastric cancer with invasion of the splenic vein and splenomegaly. The diagnosis was T4aN2M1 stage IVb gastric cancer (Fig. 4). There were no signs of liver cirrhosis. The formula applied estimated the splenic volume to be 70.3 ml, whereas the actual volume gained by the volumetric software on CT was 155 ml. Laboratory data showed a normal platelet count of 213.000/μL and normal liver function before chemotherapy. She was started on chemotherapy with trastuzumab/SOX but developed tarry stools after three courses of treatment. Upper gastrointestinal endoscopy revealed continuous bleeding from the tumor due to regrowth. Palliative radiotherapy (30 Gy in 10 fractions) was administered to control the bleeding. Hemostasis was successfully achieved. However, 2 months after starting chemotherapy, the platelet count decreased to 55.000/mm^2^, which made it difficult to resume chemotherapy. Thrombocytopenia was attributed to invasion of the splenic vein by advanced gastric cancer. PSE was performed in the same way as in case 1, with advancement of a microcatheter to the selective branch and embolization with embospheres and microcoils (Fig. 5). 60% embolization was achieved with no serious adverse events. The hospital stay was 14 days. 2 weeks after PSE, the platelet count increased from 66.000/μL to 230.000/μL.

**Figure 6:**
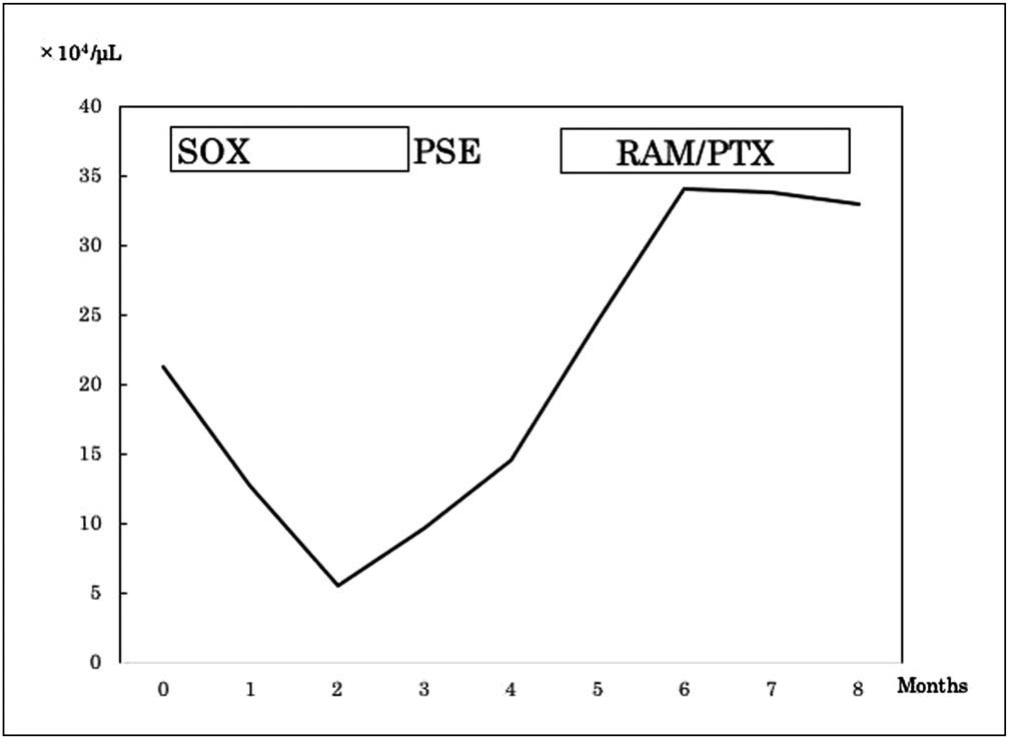
Clinical course and changes in platelet count in case 2. The platelet count increased significantly after partial splenic embolization and peaked at 230,000/mm^2^. PSE, partial splenic embolization; PTX, paclitaxel; RAM, ramucirumab; SOX, S-1 and oxaliplatin.

No thrombocytopenia was observed after PSE. Therefore, ramucirumab-paclitaxel is giving as a second-line treatment. (Fig. 6).

## 3 Discussion

This is the first report of PSE performed for hypersplenism associated with gastric cancer invading the splenic vein and continuation of chemotherapy. Causes of hypersplenism include cirrhosis, extrahepatic portal vein occlusion, portal vein thrombosis, and splenic vein invasion. Furthermore, oxaliplatin has been known to cause sinusoidal obstruction syndrome, inducing splenomegaly and formation of varices secondary to portal hypertension.^[4,5]^

Spleen volume measurements are accurately obtained whether based on the predetermined formulae can be calculated from weight and age or the volumetric software on CT.^[6]^ A volume analyzer (Synapse Vincent version 2; Fujifilm Medical Company, Tokyo, Japan) was used to measure spleen volume in our patients. In case 1, the formula applied estimated the splenic volume to be 78.3 ml; however, the actual volume was 286 ml. In case 2, the estimated volume was 70.3 ml whereas the actual volume was 155 ml. Yamaguchi et al. reported that oxaliplatin doses higher than 1040 mg/m^2^ increased the risk of sinusoidal obstruction syndrome.^[7]^ in our two cases, the doses used were 320 mg/m^2^ and 364 mg/m^2^, which are less than those previously reported.^[7–9]^ However, splenomegaly was present in both our patients before the start of treatment, and it is likely that their hypersplenism and thrombocytopenia were caused by infiltration of the tumor into the splenic vein.

Chemotherapy must be reduced or discontinued in patients with splenic hyperplasia. Patients requiring chemotherapy are often in a poor general condition due to advanced cancer, making invasive procedures difficult. However, it is important to continue chemotherapy and improve splenic hyperplasia as rapidly as possible, without complications. Treatment options for splenic hyperplasia include splenectomy and PSE. However, although splenectomy is a useful treatment for splenomegaly, it is highly invasive and has a high risk of complications.^[4]^ Moreover, infections are more likely to occur after splenectomy because of a weakened immune response. There are reports of patients who have been able to continue chemotherapy after splenectomy.^[8,9]^ However, primary resection and simultaneous resection of liver metastases were performed in addition to splenectomy, and there have been no cases in which only the spleen was resected. PSE may be an alternative treatment option for high-risk surgical candidates. Since the first report of splenic artery embolization by Maddison et al. in 1973,^[2]^ the complication rate has decreased with the use of PSE.^[3]^ PSE is indicated for hematological diseases, such as hyperplasia, idiopathic thrombocytopenic purpura, myelofibrosis, leukemia, and an increased platelet count prior to surgery or chemotherapy. PSE is considered a relatively safe procedure but has been associated with serious adverse events, including splenic abscess, acute pancreatitis, post-embolization syndrome, intravenous thrombus in the spleen, and acute gastric mucosal injury.^[10]^ To avoid these complications and obtain good results, the aim should be a splenic infarction rate of 70% to 80%.^[11]^

In both cases, chemotherapy was started with the aim of reducing tumor infiltration of the splenic vein and improving splenomegaly. However, it was difficult to continue chemotherapy due to hypersplenism. Therefore, PSE was performed with the aim of resuming chemotherapy. With a splenic infarction rate of approximately 60% to 70%, hypersplenism improved early without serious complications, and chemotherapy could be resumed and continued.

We found PSE to be a useful treatment for hypersplenism in two patients with gastric cancer. PSE could be a therapeutic strategy that allows prompt resumption and continuation of chemotherapy in such patients.

## Author contributions

**Investigation:** Taisho Hiraizumi, Fumi Naruse, Ryoya Kanda, Yuka Suzuki, Tatsuya Kakegawa, Takashi Kurosawa, Yu Yoshimasu.

**Supervision:** Toru Saguchi.

**Writing – review & editing:** Ryosuke Nakatsubo, Yoshiya Yamauchi, Atsushi Sofuni, Takao Itoi.
